# Nurses’ obesity knowledge, attitudes and practices in private facilities in Oshana, Namibia

**DOI:** 10.4102/hsag.v29i0.2385

**Published:** 2024-02-29

**Authors:** Perseverance Moyo, Rehanna Felix

**Affiliations:** 1 Department of Nursing and Midwifery, Faculty of Medicine and Health Sciences, Stellenbosch University, Stellenbosch, South Africa

**Keywords:** nurses, knowledge, practices, attitude, obesity

## Abstract

**Background:**

The prevalence of obesity has been rising globally, and this is leading to an increase in other non-communicable diseases. The level of obesity knowledge among nurses may have an impact on how they treat and perceive obese patients.

**Aim:**

The study aimed to ascertain the knowledge, attitudes and practices of nurses regarding obesity at private healthcare facilities located in Namibia’s Oshana Region.

**Setting:**

A total of four private healthcare facilities in Namibia’s Oshana area served as the study’s sites.

**Methods:**

A quantitative cross-sectional research design using proportional stratified random sampling was used to choose 250 respondents for the study. A questionnaire that respondents self-administered was used to gather the data. The reliability and validity of the questionnaire were determined during a pilot study. IBM Statistical Package for the Social Sciences (SPSS) version 27 was used for data analysis.

**Results:**

Of the participants, about two-fifths had a positive attitude (*n* = 112; 44.8%), slightly less than two-fifths had good practices (*n* = 96; 38.4%) and more than one-third had good knowledge (*n* = 97; 39%). There were differences in knowledge mean scores based on age, occupation, sex and educational level.

**Conclusion:**

The results validate the necessity for healthcare facilities to implement nurse education and mentorship initiatives, as well as to recognise and reward nurses who effectively care for patients with obesity.

**Contribution:**

This study added literature on knowledge, attitudes and practices of nurses in private healthcare facilities in Namibia, as well as factors that influence knowledge levels among the nurses.

## Introduction

Worldwide, obesity is a serious condition that receives little attention (Rogers, Young & Lotha 2021). Its prevalence has been rising in both low-to-middle-income countries and high-income countries (World Health Organization 2015). It is predicted that more than 85% of the adult population in the United States will be suffering from obesity by 2030 (Hruby & Hu 2015). Additionally, between 1993 and 2009, China’s adult male population’s prevalence of obesity increased four times. In certain European countries, obesity prevalence rose by roughly 30% during the previous 10 years (Agha & Agha 2017). Because obesity can result in a wide range of comorbidities, it could be detrimental to the public health system (Lobstein 2014). According to Kyrou et al. (2017), obesity is also linked to severe disability, early death and treatment failure in some illnesses such as diabetes mellitus, hypertension and other cardiovascular diseases. Furthermore, obesity places a significant financial strain on people, families and countries around the world (Heckers et al. 2022; Okunogbe et al. 2021).

The treatment of obesity by nurses and their perceptions of obese patients may be influenced by their knowledge of obesity (Gormley & Melby 2020). Highly informed nurses are more likely to recognise that patients are not solely to blame for their obesity, which may have an impact on their attitudes and practices (Gormley & Melby 2020). A positive attitude about obesity among nurses is linked to a greater desire to learn more about the illness, which could improve their practices in managing obesity and how they treat obese patients (Wynn et al. 2018). The way that nurses approach obesity is determined by a variety of issues, including the nurses’ knowledge of obesity, their attitudes towards obese patients and the patients’ attitudes towards them, as well as organisational variables like working hours (Nolan et al. 2012).

Strategies and policies for lowering obesity at multiple levels are necessary to make a noticeable impact because of the complexity of the obesity pandemic (Malik, Willet & Hu 2013). Given their critical role in managing obesity, nurses ought to be trained in treating obesity. Training should also emphasise working with multidisciplinary teams and using behavioural strategies to effectively combat the obesity pandemic (Bray et al. 2016; Stephenes, Cobiac & Veerman 2014).

## Study aim and objectives

This study aimed to determine the nurses’ levels of obesity knowledge, attitudes and practices in private healthcare facilities in the Oshana Region, Namibia. The study’s specific goals were to ascertain the:

- degree to which nurses working in private healthcare facilities are knowledgeable about obesity;
- nurses’ attitudes on obesity;
- nurses’ practices regarding obesity;
- association between means of total knowledge scores of nurses and their sociodemographic characteristics;
- association between nurses’ knowledge scores and attitude scores, knowledge scores and practice scores and attitude scores and practice scores regarding obesity;
- association between nurses’ knowledge of obesity and their sociodemographic characteristics.

## Research methods and design

### Research design

The knowledge, attitudes and practices of nurses towards obesity were assessed using a quantitative cross-sectional survey.

### Study setting and population

The study was conducted in the Oshana Region, the most populous of Namibia’s 14 regions. The majority of the nurses who work in the private sector in the Oshana Region work in the four private healthcare facilities where the study was carried out. All 400 nurses who worked at the four selected private healthcare facilities made up the study population.

### Sample size and sampling

The sample size was calculated using Yamane’s formula. The formula is as follows: n = N/(1+e^2^N), where n is the sample size, N is the population size and e is the margin of error. The margin of error used for the study was 5% because the researcher wanted a confidence level of 95%. Using a population size of 400, the sample size for this study was found to be 250. To choose the participants, a proportional stratified random sampling method was used. One hospital (OPH) had 150 nurses (37.5% of the total population), MCO had 101 (25.25%), BMC had 80 (20%) and OHC had 69 (17.25%). All nurses were listed according to their places of employment in the first step of the sampling process, and they were then chosen using a simple random sampling method in the second step. The total number of participants selected for each hospital was 94 for OPH, 63 for MCO, 50 for BMC and 43 for OHC.

### Inclusion and exclusion criteria

Nurses who were employed full time at the four healthcare facilities where the study was carried out were included in this study. Nurses who were absent or on vacation on the days the data were gathered were not included in the study.

### Data collection instrument

The data were gathered using a questionnaire with four sections. The participants’ sociodemographic details, including age, sex, knowledge of body mass index (BMI), highest level of education and occupation, were covered in the first section. The knowledge, attitudes and practices of the nurses addressing obesity were assessed using statements with five Likert scale responses included in the other three parts. The questionnaire is attached as Supplementary File 1.

### Reliability of the questionnaire

Before the data were collected, the questionnaire’s reliability was established in a pilot study. The stability of the questionnaire was evaluated using the test-retest method, and its internal consistency was evaluated using Cronbach’s alpha. The questionnaire was administered to the pilot test participants twice, 1 week apart from one another. The pilot study was carried out at a private healthcare facility that was not chosen for the main study. Forty-six nurses were selected from all the wards at the institution using a convenience sampling method. IBM Statistical Package for the Social Sciences (SPSS) version 27 for Windows was used to analyse the data, and the correlation coefficient came out to be 0.85. The knowledge score items had a Cronbach’s alpha of 0.82, the attitude score items of 0.87 and the practice score items of 0.80.

### Validity of the questionnaire

During the pilot study, the questionnaire’s face validity was evaluated. The readability, uniformity of style and arrangement, as well as the questions and clarity of the language clarity used, were all evaluated by the participants. A judgemental approach was used to evaluate the questionnaire’s content validity. This required consulting obesity experts as well as reviewing the literature on the subject. The consulted obesity experts included two physicians and two dieticians, who all agreed that the questionnaire was valid.

### Recruitment process and data collection

The researcher and two research assistants who were trained by the researcher before data collection met the nurses in the wards where they work and described the study’s aim and objectives. They then asked nurses who had been chosen at random via Microsoft Excel to participate in the research. The nurses who decided to take part were asked to sign an informed consent and given the questionnaire to complete on their own. Data were collected in October 2021.

### Data management and analysis

Precoded responses were entered into SPSS. Frequencies and percentages were used to report the analysis results of both nominal and ordinal data. The total scores of each respondent’s answers to the knowledge, attitudes and practices statements in the questionnaire were added to determine the nurses’ knowledge, attitudes and practices about obesity. The three categories that were created based on the total scores were poor, average and good. If a participant’s total score was between 40 and 50, it was considered strong knowledge; if it was between 30 and 39, it was considered average knowledge, and if it was less than 30, it was considered low knowledge. Participants who scored between 15 and 19 were deemed to have average attitudes and practices, those who scored under 15 were seen to have poor attitudes and practices, and those whose total score fell between 20 and 25 were deemed to have good attitudes and practices. To find out whether there were any variations in the knowledge scores’ means depending on sex or individual BMI knowledge, independent t-tests were used. Furthermore, one-way ANOVA tests were executed to determine whether the means of the knowledge scores differed according to occupation or level of education. Pearson’s correlation coefficients were used to ascertain the associations between knowledge and attitude, knowledge and practice and attitude and practice. Chi-square tests and logistic regression were used to assess the relationships between the levels of knowledge and the sociodemographic characteristics of the respondents. The 95% confidence intervals and *p*-values of less than 0.05 from the chi-square test were utilised to assess the findings’ statistical significance.

### Ethical considerations

The study obtained ethical approval from a Stellenbosch University’s Health Research Ethics Committee (S21/06/103), as well as institutional permissions from the private healthcare institutions where the study was conducted.

## Results

All the selected respondents completed the questionnaire (*n* = 250, 100%). The age group of 31–35 years had around one-fifth of the respondents (*n* = 51; 20.4%), while the age group of 51–55 years included the fewest respondents (*n* = 13; 5.2%). The majority of the respondents were females (*n* = 162; 65%), while a few were males (*n* = 88; 35%). The majority of the respondents (*n* = 144; 57.6%) had their highest educational level as a diploma, while a few (*n* = 3; 1.2%) had doctorate degrees. Most of the respondents were registered nurses (*n* = 150; 60%), while a few were nurse managers (*n* = 25; 10%). Few respondents (*n* = 51; 20%) knew their BMI, while the majority (*n* = 199; 80%) did not know their BMI. More details are presented in Table 1.

**TABLE 1:** Frequency distribution of sociodemographic characteristics (*N* = 250).

Characteristic	*n*	%
**Age (years)**		
20–25	45	18.0
26–30	46	18.4
31–35	51	20.4
36–40	35	14.0
41–45	40	16.0
46–50	20	8.0
51–55	13	5.2
**Occupation**		
Enrolled nurse	75	30.0
Registered nurse	150	60.0
Nurse manager	25	10.0
**Sex**		
Male	88	35.2
Female	162	64.8
**Personal BMI knowledge**		
Yes	51	20.4
No	199	79.6
**Education**		
Diploma	144	57.6
Bachelor’s degree	95	38.0
Master’s degree	8	3.2
Doctorate degree	3	1.2

BMI, body mass index.

## Knowledge of nurses regarding obesity

### Frequency distribution of responses to knowledge statements

Overall, the respondents’ responses to each of the 10 statements were statistically significantly different. The majority of respondents gave the right answers to the 10 statements. Most respondents (*n* = 184; 73.6%) indicated that obesity is diagnosed using BMI and most (*n* = 164; 65.6%) indicated that an individual with BMI above 30 is considered obese. Almost one-half of the respondents (*n* = 122; 48.8%) stated that stigma associated with obesity may cause mental health issues in patients with obesity. Most of the respondents (*n* = 173; 69.2%) knew that there is a link between obesity and some non-communicable diseases, most of the respondents (*n* = 176; 70.4%) were aware that obesity is a risk factor for several cancers, and most of the respondents (*n* = 170; 68%) were aware that increasing daily exercise reduces obesity. In addition, most of the respondents (*n* = 162; 64.8%) understood that eating more fruits and vegetables can help reduce obesity, and most of the respondents (*n* = 163; 65.2%) knew that eating less at meals can help reduce obesity. Most respondents (*n* = 157; 62.8%) agreed that obesity can raise healthcare costs. More details are presented in Table 2.

**TABLE 2:** Frequency distribution of responses to knowledge statements.

Statement	Total		One-sample chi-square *p*-value
	*n*	%	
**Obesity is diagnosed using body mass index (BMI)**	**-**	**-**	***p* < 0.01**
Strongly disagree	24	9.6	**-**
Disagree	24	9.6	**-**
Do not know	18	7.2	**-**
Agree	70	28	**-**
Strongly agree	114	45.6	**-**
**An individual with BMI above 30 is considered obese**	**-**	**-**	***p* < 0.01**
Strongly disagree	22	8.8	**-**
Disagree	36	14.4	**-**
Do not know	28	11.2	**-**
Agree	73	29.2	**-**
Strongly agree	91	36.4	**-**
**Obesity can be reduced by eating less during meals**	**-**	**-**	***p* < 0.01**
Strongly disagree	25	10.0	**-**
Disagree	31	12.4	**-**
Do not know	31	12.4	**-**
Agree	73	29.2	**-**
Strongly agree	90	36.0	**-**
**Eating more fruits and vegetables can help reduce obesity**	**-**	**-**	***p* < 0.01**
Strongly disagree	24	9.6	**-**
Disagree	33	13.2	**-**
Do not know	31	12.4	**-**
Agree	78	31.2	**-**
Strongly agree	84	33.6	**-**
**Obesity can increase the cost of healthcare**	**-**	**-**	***p* < 0.01**
Strongly disagree	31	12.4	**-**
Disagree	38	15.2	**-**
Do not know	24	9.6	**-**
Agree	59	23.6	**-**
Strongly agree	98	39.2	**-**
**Doing more exercises daily reduces obesity**	**-**	**-**	***p* < 0.01**
Strongly disagree	23	9.2	**-**
Disagree	26	10.4	**-**
Do not know	31	12.4	**-**
Agree	67	26.8	**-**
Strongly agree	103	41.2	**-**
**Obesity is associated with certain non-communicable diseases**	**-**	**-**	***p* < 0.01**
Strongly disagree	23	9.2	**-**
Disagree	30	12.0	**-**
Do not know	24	9.6	**-**
Agree	75	30.0	**-**
Strongly agree	98	39.2	**-**
**Obesity is a risk factor for certain cancers**	**-**	**-**	***p* < 0.01**
Strongly disagree	16	6.4	**-**
Disagree	41	16.4	**-**
Do not know	17	6.8	**-**
Agree	73	29.2	**-**
Strongly agree	103	41.2	**-**
**Management of obesity involves combining different strategies like drugs, surgery, psychotherapy and behavioural therapy**	**-**	**-**	***p* < 0.01**
Strongly disagree	15	6.0	**-**
Disagree	19	7.6	**-**
Do not know	33	13.2	**-**
Agree	77	30.8	**-**
Strongly agree	106	42.4	**-**
**Obesity stigma may lead to mental health problems among obese patients**	**-**	**-**	***p* < 0.01**
Strongly disagree	21	8.4	**-**
Disagree	30	12.0	**-**
Do not know	22	8.8	**-**
Agree	55	22.0	**-**
Strongly agree	122	48.8	**-**

Bold values represent statistically significant findings.

### Total knowledge scores and frequency distribution of knowledge levels of respondents

**FIGURE 1:**
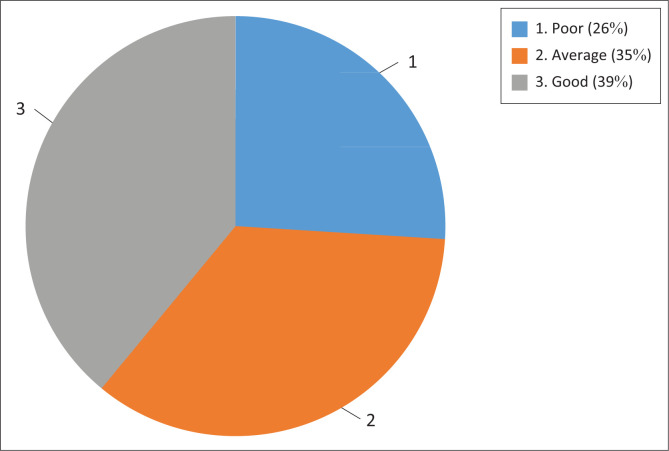
Frequency distribution of knowledge levels of respondents.

The mean of the total knowledge scores was 37.85 (*s.d*. = 7.96). The respondents’ lowest score was 25, and their highest score was 50. Nearly two-fifths of the respondents (*n* = 97; 39%) had high obesity knowledge, whereas about a quarter (*n* = 65; 26%) had poor knowledge. More details on the frequency distribution of knowledge levels are presented in Figure 1.

## Attitudes of nurses regarding obesity

### Frequency distribution of responses to attitude statements

Overall, the respondents’ responses to each of the five statements were statistically significantly different. The majority of the respondents gave correct responses to the five statements. About two-thirds of the respondents (*n* = 169; 67.6%) were aware that obesity is a medical condition, most (*n* = 171; 68.4%) indicated that nurses should be exemplary and try to have a normal weight, and almost two-thirds (*n* = 167; 66.8%) thought that caring for obese patients was personally rewarding. Additionally, about seven-tens of the respondents (*n* = 177; 70.8%) understood that not all obese patients gain their weight due to negligence, and most (*n* = 178; 71.2%) stated that individuals who are obese are not inherently lazier than those of normal weight. More details are presented in Table 3.

**TABLE 3:** Frequency distribution of responses to attitude statements.

Statement	Total		One-sample chi-square test summary
	*n*	%	*p*-value
**Obesity is a medical condition**	**-**	**-**	***p* < 0.01**
Strongly disagree	28	11.2	**-**
Disagree	36	14.4	**-**
Do not know	17	6.8	**-**
Agree	63	25.2	**-**
Strongly agree	106	42.4	**-**
**Nurses should set an example and strive to maintain normal weight**	**-**	**-**	***p* < 0.01**
Strongly disagree	19	7.6	**-**
Disagree	38	15.2	**-**
Do not know	22	8.8	**-**
Agree	70	28.0	**-**
Strongly agree	101	40.4	**-**
**Treating obese patients is professionally gratifying**	**-**	**-**	***p* < 0.01**
Strongly disagree	23	9.2	**-**
Disagree	30	12.0	**-**
Do not know	30	12.0	**-**
Agree	70	28.0	**-**
Strongly agree	97	38.8	**-**
**Not all obese patients develop obesity through carelessness**	**-**	**-**	***p* < 0.01**
Strongly disagree	21	8.4	**-**
Disagree	35	14.0	**-**
Do not know	17	6.8	**-**
Agree	76	30.4	**-**
Strongly agree	101	40.4	**-**
**Obese patients are not lazier than normal weight people**	**-**	**-**	***p* < 0.01**
Strongly disagree	22	8.8	**-**
Disagree	34	13.6	**-**
Do not know	16	6.4	**-**
Agree	72	28.8	**-**
Strongly agree	106	42.4	**-**

Bold values represent statistically significant findings

### Total attitude scores and frequency distribution of attitude levels of respondents

**FIGURE 2:**
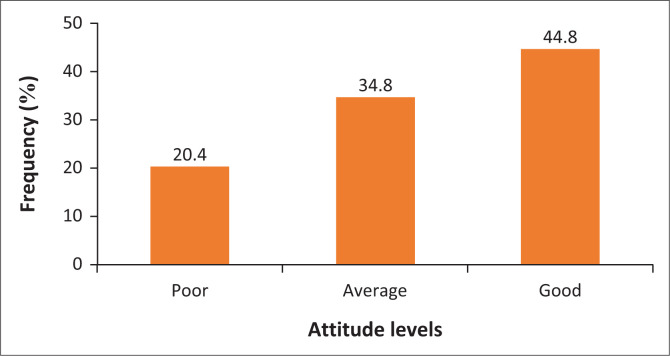
Frequency distribution of attitude levels of respondents.

The mean of the total attitude scores was 18.92 (*s.d*. = 4.37). The respondents’ lowest score was 10, and their highest score was 25. Almost half of the respondents (*n* = 112; 44.8%) had a positive attitude, whereas about 20% of the respondents (*n* = 65; 20.4%) had a negative attitude. More details on the frequency distribution of attitude levels are presented in Figure 2.

## Practices of nurses regarding obesity

### Frequency distribution of responses to practice statements

Overall, the respondents’ responses to each of the five statements were statistically significantly different. Most respondents responded correctly to each of the five statements. Most of the respondents (*n* = 162; 64.8%) stated that they treated obesity like any other condition, about three-fifths (*n* = 154; 61.6%) stated that they calculated BMI for all their patients, and most (*n* = 154; 61.6%) also stated that they always urged obese patients to lose weight. In addition, most of the respondents (*n* = 180; 72%) specified that they always provided nutritional advice to their obese patients, and most (*n* = 171; 68.4%) said they spent time talking to all of their obese patients about the risks of obesity. More details are presented in Table 4.

**TABLE 4:** Frequency distribution of responses to practice statements.

Statement	Total		One-sample chi-square test summary
	*n*	%	*p*-value
**I treat obesity like any other condition**	**-**	**-**	***p* < 0.01**
Strongly disagree	25	10.0	**-**
Disagree	43	17.2	**-**
Do not know	20	8.0	**-**
Agree	68	27.2	**-**
Strongly agree	94	37.6	**-**
**I calculate BMI for all my patients**	**-**	**-**	***p* < 0.01**
Strongly disagree	30	12.0	**-**
Disagree	45	18.0	**-**
Do not know	21	8.4	**-**
Agree	62	24.8	**-**
Strongly agree	92	36.8	**-**
**I always encourage obese patients to lose weight**	**-**	**-**	***p* < 0.01**
Strongly disagree	28	11.2	**-**
Disagree	41	16.4	**-**
Do not know	27	10.8	**-**
Agree	68	27.2	**-**
Strongly agree	86	34.4	**-**
**I always advise on diets to all my obese patients**	**-**	**-**	***p* < 0.01**
Strongly disagree	17	6.8	**-**
Disagree	33	13.2	**-**
Do not know	20	8.0	**-**
Agree	86	34.4	**-**
Strongly agree	94	37.6	**-**
**I always take time to discuss the dangers of obesity to all my obese patients**	**-**	**-**	***p* < 0.01**
Strongly disagree	19	7.6	**-**
Disagree	33	13.2	**-**
Do not know	27	10.8	**-**
Agree	71	28.4	**-**
Strongly agree	100	40.0	**-**

BMI, body mass index.

Bold values represent statistically significant findings.

### Total practice scores and frequency distribution of practice levels of respondents

**FIGURE 3:**
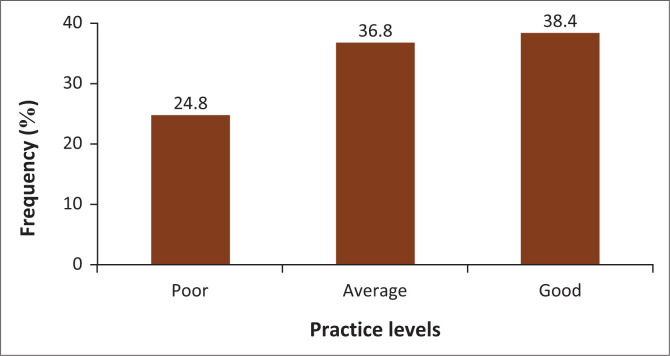
Frequency distribution of practice levels of respondents.

The mean score for overall practice was 18.42 (*s.d*. = 4.21). The respondents’ lowest score was 10, and their highest score was 25. About 38% of the respondents (*n* = 96; 38.4%) had good obesity practices, while about a quarter (*n* = 62; 24.8%) had poor practices. More details on the frequency distribution of practice levels are presented in Figure 3.

### Correlation between knowledge, attitudes and practices of respondents

Total knowledge scores strongly positively correlated with total attitude scores (r = 0.812, *p* < 0.01), total knowledge scores strongly positively correlated with total practice scores (r = 0.812, *p* < 0.01) and total attitude scores also had a strong positive correlation with total practice scores (r = 0.834, *p* < 0.01).

### Association between the means of knowledge scores and sociodemographic characteristics

The males’ total knowledge scores’ mean (*M* = 39.26, *s.d*. = 8.57) was statistically significantly greater than that of females (*M* = 37.09, *s.d*. = 7.82), (t [*df* 248] = 2.077, *p* = 0.039). In contrast, the total knowledge scores’ mean for respondents who knew their BMI (*M* = 39.10, *s.d*. = 7.11) was not statistically significantly different from that of those who did not know their BMI (*M* = 37.53, *s.d*. = 8.15), (t [*df* 248] = 1.255, *p* = 0.211). The total knowledge scores’ mean statistically significantly varied by the educational level of the respondents (*F* [3, 246] = 16.64, *p* < 0.01). Additionally, the results show that the total knowledge scores’ mean of the respondents statistically significantly differed by occupation (*F* [2, 247] = 34.56, *p* < 0.01).

### Association between knowledge levels and sociodemographic characteristics

The chi-square tests showed a statistically significant association between age groups and the respondents’ obesity knowledge, χ^2^ (*df* = 5, *n* = 250) = 12.36, *p* = 0.03; between education and obesity knowledge, χ^2^ (*df* = 2, *n* = 250) = 39.98, *p* < 0.01 and between occupation and obesity knowledge, χ^2^ (*df* = 2, *n* = 250) = 17.62, *p* < 0.01. The correlation between sex and obesity knowledge was not statistically significant, χ^2^ (*df* = 1, *n* = 250) = 2.53, *p* = 0.11; nor was there a correlation between personal BMI awareness and obesity knowledge, χ^2^ (*df* = 1, *n* = 250) = 0.51, *p* = 0.48.

The likelihood of having good obesity knowledge was statistically significantly less for age groups 20–25 years and 26–30 years compared to the age group 46–55 years, crude odds ratio (OR) = 0.31, 95% CI (0.12–0.79) and OR = 0.37, 95% CI (0.14–0.92), respectively. Age groups 31–35, 36–40 and 41–45 years exhibited levels of knowledge that did not statistically significantly vary from age group 46–55 years, OR = 0.50, 95% CI (0.20–1.21), OR = 0.44, 95% CI (0.16–1.16) and OR = 1.02, 95% CI (0.40–2.57), respectively. Compared to nurse managers, enrolled nurses had a statistically significant lower likelihood of having a good knowledge level of obesity, OR = 0.15, 95% CI (0.06–0.41). Registered nurses’ level of obesity knowledge did not statistically significantly vary with that of nurse managers, OR = 0.43, 95% CI (0.18–1.04). Respondents with diplomas had a statistically significantly lower likelihood of having a good knowledge level of obesity when compared with respondents with master’s and doctorate degrees, OR = 0.11, 95% CI (0.03–0.43). Between males and females, there was no difference in knowledge of obesity, OR = 1.54, 95% CI (0.90–2.61). Furthermore, there was no significant variation in respondents’ knowledge of obesity between respondents with bachelor’s degrees and those with master’s and doctoral degrees, OR = 0.56, 95% CI (0.14–2.26). More details are presented in Table 5.

**TABLE 5:** Association between knowledge levels and sociodemographic characteristics of respondents.

Characteristics	Crude odds ratios	95% CI*	Chi-square test *p*-value
**Age (years)**	**-**	**-**	***p* = 0**.03
20–25	**0.31**	**0.12–0.79**	-
26–30	**0.37**	**0.14–0.92**	-
31–35	0.50	0.20–1.21	-
36–40	0.44	0.16–1.16	-
41–45	1.02	0.40–2.57	-
46–55	Reference	Reference	-
**Sex**	**-**	**-**	*p* = 0.11
Male	1.54	0.90–2.61	-
Female	Reference	Reference	-
**Occupation**	**-**	**-**	***p* < 0.01**
Enrolled nurse	**0.15**	**0.06–0.41**	-
Registered nurse	0.43	0.18–1.04	-
Nurse manager	Reference	Reference	-
**Education**	**-**	**-**	***p* < 0.01**
Diploma	**0.11**	**0.03–0.43**	-
Bachelor’s degree	0.56	0.14–2.26	-
Master’s and doctorate degrees	Reference	Reference	-
**Knowledge of personal BMI**	**-**	**-**	*p* = 0.48
Yes	1.25	0.67–2.34	-
No	Reference	Reference	-

BMI, body mass index; CI, confidence interval.

Bold values represent statistically significant findings.

## Discussion

The findings of this study indicate that respondents’ knowledge of obesity was average. In the current study, the knowledge scores’ mean was greater than it was in a study conducted in the United Kingdom (Wynn et al. 2018). The occupation, age, sex and level of education all affected the mean knowledge score. Nonetheless, the individuals’ awareness of their BMI had no bearing on the mean score for knowledge. These results are similar to the results of research conducted in China and Iraq, which found that nurses’ understanding of obesity was influenced by their educational background (Fan et al. 2020; Tiryag & Atiyah 2021). The differences in the mean knowledge score based on the educational backgrounds of the participants may have been caused by more knowledge about obesity among nurses with higher education levels. According to the findings, male nurses scored higher on knowledge tests than female nurses. This was expected given that more men than women in this study had earned bachelor’s, master’s or doctoral degrees.

In contrast to a Malaysian study (Ardzi et al. 2014), a lower percentage of respondents in this study were aware that obesity can be diagnosed using BMI. Additionally, a lower percentage of respondents in this study replied correctly to the statement ‘Eating more fruits and vegetables can help reduce obesity’, compared to those in a study carried out in Switzerland (Bucher Della Torre et al. 2018). Because they care for less obese patients than those in the Swiss study, nurses in this study may have had less exposure to obesity, which could account for the findings (Bucher Della Torre et al. 2018). A higher percentage of respondents in this study had good obesity knowledge compared to those in studies conducted in the United Kingdom and Iraq (Gormley & Melby 2020; Tiryag & Atiyah 2021). This higher proportion of nurses who had good knowledge in this study might be because the nurses worked at private healthcare facilities where nurses manage more obese patients compared to public healthcare facilities. The nurses’ understanding of obesity may have increased as a result of their desire to learn more about the condition after managing more obese patients (Dietz et al. 2015).

This study’s results depicted that the respondents’ obesity attitudes were average. In contrast to a Swiss study (Bucher Della Torre et al. 2018), the mean attitude score in this study was higher. The literature review findings were at odds with the higher total attitude scores in this study compared to those from the study carried out in Switzerland, as respondents in the Swiss had higher total knowledge scores (Bucher Della Torre et al. 2018). Respondents with higher levels of obesity knowledge are anticipated to have more favourable views towards patients with obesity (Wynn et al. 2018). In this study, the mean attitude score did not vary by sex or personal BMI knowledge, but it varied by educational attainment and occupation. These results are consistent with those of a study done in Germany, which found that respondents’ views about obesity were influenced by their level of education and employment (Sikorski et al. 2013). Compared to nurses in a prior study (Teixeira, Paias-Ribeiro & Pinho da Costa Maia 2012), more nurses in this study strongly agreed that caring for obese patients was professionally satisfying. This finding may be attributable to the respondents’ higher knowledge of obesity and hence more willingness to help obese patients.

The practice scores’ mean in this study was greater than the one revealed in a British study (Zhu, Norman & While 2013). The fact that the respondents in this study had a higher knowledge of obesity may help to explain this finding. The mean practice score in this study varied by education and occupation but not by sex or awareness of one’s own BMI. The percentage of respondents who strongly agreed that they treated obesity like any other condition in this study was slightly lower than that reported in a study in Pakistan (Kausar, Mukhtar & Shaheen 2021). In contrast to what was found in a study conducted in the United States (Petrin et al. 2017), a lower proportion of respondents in this study strongly agreed that they took the time to explain the risks of obesity to their obese patients. These results are consistent with the respondents’ low obesity practice levels in this study. This study revealed that a lower proportion of respondents had good obesity practices compared to the results of a South African study (Van Tonder, Kelly & Van Rooyen 2021), possibly because they also have lower obesity knowledge compared to those in the South African study.

The results of this study depict a significant correlation between knowledge scores and attitude scores, knowledge scores and practice scores and attitude scores and practice scores. These results are comparable to the findings of a study conducted in the United Kingdom (Wynn et al. 2018). The results of this study also lend credence to the conceptual framework, which proposed that if nurses’ attitudes about obesity change, so will their practices (Pearce et al. 2019). The findings of this study showed a relationship between respondents’ knowledge of obesity and their age, occupation and level of education. Nevertheless, no relationship was found between knowledge and sex or knowledge of personal BMI. The results of this study concur with those of studies conducted in China and Iraq, which also revealed a relationship between obesity knowledge and education (Fan et al. 2020; Tiryag & Atiyah 2021). The findings of this study are similar to those of a study done in Iraq, which found no relationship between knowledge of obesity and either sex or personal BMI (Tiryag & Atiyah 2021). However, the findings of this study were different from those of an Iraqi study since a relationship between knowledge of obesity and age was discovered while this association was not seen in the Iraqi study (Tiryag & Atiyah 2021). The study’s findings are believable because participants’ ages and educational attainment were correlated. As education is acknowledged to increase knowledge about obesity, it would follow that age would increase knowledge about obesity because older persons often had better educational levels. The findings of this study are in agreement with the findings of other studies conducted elsewhere in the world. Considering this, we believe that our findings are generalisable to nurses working at similar healthcare facilities in the region and globally.

## Study limitations

The study’s main limitation is that because it was only done in private healthcare facilities and one region of Namibia, its findings cannot be extrapolated to the entire country. Furthermore, the study did not control outside factors like training and media campaigns that could have altered study participants’ levels of knowledge.

## Recommendations

### Training on obesity

All nurses should have access to training opportunities to increase their understanding of obesity. This can be done by providing consistent in-service training on obesity at healthcare facilities, paying for nurses to register for courses on obesity that are recognised internationally or to attend academic conferences, and advocating for ongoing revisions to the training programmes and curricula for nurses so that obesity can be given priority.

### Offering rewards to nurses

Private healthcare facilities should provide some incentives to those who are considered to have good attitudes and practices by patients. Patients can be requested to vote anonymously for their favourite nurses every month. This may motivate nurses to learn more about obesity that may result in a change in their attitudes and practices concerning obesity.

### Mentoring newly qualified nurses

The findings of the study suggest that nurses who were older had a greater understanding of obesity, hence private healthcare facilities should launch mentorship initiatives for recently qualified nurses. As freshly qualified nurses acquire skills while executing their jobs, mentoring new nurses may be less expensive. This may reduce recruitment costs, because the mentored nurses may become knowledgeable enough to deliver obesity services, which may have needed specially trained nurses. Such mentoring may help increase newly qualified nurses’ confidence in managing obese patients.

### The correct blending of nurses

There are different levels of knowledge, attitudes and practices regarding obesity among nurses of different ages and educational levels. Therefore, nurses should be grouped in such a way that there are different ages and educational levels in the groups. This may assist with the transfer of knowledge among them, resulting in an improvement of knowledge, attitudes and practices among nurses who have poor levels.

## Conclusion

The proportion of respondents in this study who had good knowledge, attitudes and practices was low. There were significant positive correlations between knowledge and attitude, knowledge and practice and attitude and practice scores. In addition, there were relationships between obesity knowledge and age, occupation and education. It is therefore important that nurses be trained on obesity and rewarded for good attitudes and practices regarding obesity. Furthermore, healthcare facilities should mentor newly qualified nurses on obesity and blend nurses according to education and age groups.

## Appendix 1

### Supplementary file 1: Questionnaire

**Table:** Screening questions

**Do you work full-time at this health care institution?** No Yes **Have you had formal training in a health-related field?** No Yes **Are you involved in the day to day caring of obese patients?** No Yes

If your answer is yes to all the above questions, then please complete the rest of the questionnaire.

### Section A: Demographic profile of participants

Please put a cross (x) in the blank box in front of the statement you have chosen as your answer.

1. How old are you?
  □ 20–25     □ 26–30     □ 31–35     □ 36–40     □ 41–45     □ 46–50     □ 51–55
2. What is your sex?
  □ Male     □ Female
3. Do you know your BMI?
  □ Yes     □ No
4. What is the highest educational level you attained?
  □ Diploma     □ Bachelor’s degree     □ Master’s degree     □ Doctorate
5. What is your occupation?
  □ Nurse Unit Manager     □ Registered Nurse     □ Enrolled Nurse

### Section B: Knowledge of nurses regarding obesity

**Table:** Please choose one response on each statement and show it by a cross (x). Please make sure that you do not consult anyone or any other sources when answering these questions.

	Statement	Strongly disagree	Disagree	Do not know	Agree	Strongly agree
	**Score**	1	2	3	4	5
6.	Obesity is diagnosed using body mass index (BMI).					
7.	An individual with BMI above 30 is considered obese.					
8.	Obesity can be reduced by eating less during meals.					
9.	Eating more fruits and vegetables can help reduce obesity.					
10.	Obesity can increase the cost of health care.					
11.	Doing more exercises daily reduces obesity.					
12.	Obesity is associated with some non-communicable diseases.					
13.	Obesity is a risk factor for some cancers.					
14.	Management of obesity involves combining different strategies like drugs, surgery, psychotherapy and behavioural therapy.					
15.	Obesity stigma may lead to mental health problems among obese patients.					

### Section C: Attitudes of nurses regarding obesity

**Table:** Please choose one response for each source and show your preferred response by a cross (x) in the provided boxes.

	Statement	Strongly disagree	Disagree	Do not know	Agree	Strongly agree
	**Score**	1	2	3	4	5
16.	Obesity is a medical condition.					
17.	Nurses should set an example and strive to maintain normal weight.					
18.	Treating obese patients is professionally gratifying.					
19.	Not all obese patients develop obesity through carelessness.					
20.	Obese patients are not lazier than normal weight people.					

### Section D: Practices of nurses regarding obesity

**Table:** Please choose one response for each source and show your preferred response by a cross (x) in the provided boxes.

	Statement	Strongly disagree	Disagree	Not sure	Agree	Strongly agree
	**Score**	1	2	3	4	5
21.	I treat obesity like any other condition.					
22.	I calculate BMI for all my patients.					
23.	I always encourage obese patients to lose weight.					
24.	I always give advice on diet to all my obese patients.					
25.	I always take time to discuss the dangers of obesity to all my obese patients.					
